# Organo-fluorine chemistry V

**DOI:** 10.3762/bjoc.17.63

**Published:** 2021-03-17

**Authors:** David O’Hagan

**Affiliations:** 1 School of Chemistry, Biomedical Sciences Research Complex, University of St Andrews, North Haugh, St Andrews, Fife KY16 9ST, United Kingdom

**Keywords:** Organo-fluorine chemistry

This is the fifth thematic issue on organo-fluorine chemistry in the *Beilstein Journal of Organic Chemistry* that I have had the pleasure to edit, and these themed issues have gone progressively from strength to strength ever since the first was assembled in 2008. In this issue 45 papers are collected, a record for a thematic issue in the Journal, and there is no doubt that the *Beilstein Journal of Organic Chemistry* has become a settled home for papers in contemporary organo-fluorine chemistry. Authors have presented their recent findings or, in some cases, reviewed important areas, from bioactives to materials, and collectively the papers highlight the vibrancy and range of activity associated with this theme. Organo-fluorine chemistry merits our attention as an important discipline within contemporary organic chemistry contributing in a unique way to innovation and performance in the development of functional molecules.

This issue will be historically memorable as all of the manuscripts were submitted through the covid-19 virus lockdown period which impacted universities and research laboratories across the world. As Guest Editor I am indebted to all of the authors for maintaining their commitment to contribute through what was a particularly difficult and unusual period of professional life.

I thank the referees who gave of their time, and also the outstanding level of professionalism of the Editorial and Production Team at the *Beilstein Journal of Organic Chemistry* for their continuous attention to detail and the quality of the final published copy. The papers appear as contributions to the Journal in the normal sense, but they are also conveniently collected here as a single document.

David O’Hagan

St Andrews, Scotland, February 2021

